# Potential Clinical Applications of Ozone Therapy in Dental Specialties—A Literature Review, Supported by Own Observations

**DOI:** 10.3390/ijerph20032048

**Published:** 2023-01-22

**Authors:** Izabela Barczyk, Diana Masłyk, Natalia Walczuk, Karina Kijak, Piotr Skomro, Helena Gronwald, Maria Pawlak, Angelika Rusińska, Natalia Sadowska, Barbara Gronwald, Adam Andrzej Garstka, Danuta Lietz-Kijak

**Affiliations:** 1 Student Scientific Society at the Department of Propaedeutic, Physical Diagnostics and Dental Physiotherapy, Faculty of Medicine and Dentistry, Pomeranian Medical University, 70-204 Szczecin, Poland; 2 Department of Propaedeutic, Physical Diagnostics and Dental Physiotherapy, Faculty of Medicine and Dentistry, Pomeranian Medical University, 70-204 Szczecin, Poland; 3 Doctoral Studies at the Department of Propaedeutic, Physical Diagnostics and Dental Physiotherapy, Faculty of Medicine and Dentistry, Pomeranian Medical University, 70-204 Szczecin, Poland

**Keywords:** ozone, ozone therapy, therapeutic applications, oxidants, dental disinfectants, oral medicine, physical therapy in dentistry

## Abstract

Apart from conventional treatment, dentists are increasingly relying on physical therapy modalities in their clinical practice. The aim of this literature review is to analyze the clinical relevance and potential uses of ozone in modern dentistry. The research question is geared towards detailing the multiple potential applications of ozone therapy in a range of dental specialties. Based on the available literature, accessed via the PubMed, Google Scholar, Scopus, and EBSCO databases, a detailed search of the electronic literature was performed for 2001–2022. Eligible studies were chosen according to inclusion and exclusion criteria, using keywords: ozone, ozone therapy, therapeutic applications, oxidants, dental disinfectants, oral medicine, physical therapy in dentistry. Out of 834 manuscripts, 273 studies were curated. A total of 70 publications were used in the final consideration. After assessing their quality, they were analyzed to determine the relevance and potential use of ozone in the various aspects of modern dentistry. Ozone therapy is used mainly as an adjunct to the primary clinical or pharmacological treatment. In some cases of oral mucosal disease, it has proven effective as a primary therapy. During the literature analysis, it was noted that ozone therapy in dentistry is a subject of ongoing research, and the results are not always consistent. The multitude of studies in the literature on the applications of ozone in dentistry reflects the search for its undiscovered physical therapeutic potential.

## 1. Introduction

The element oxygen exists in two allotropic forms. In addition to the most common diatomic oxygen (O_2_), there is a triatomic variant (O_3_, ozone), which is made up of three atoms joined by two identical oxygen–oxygen bonds with a length of 0.1278 ± 0.003 nm and an angle of 116° and 49′ ± 30″ between them. Its molecular weight is 47.98 g/mol. Ozone is thermodynamically highly unstable and, depending on temperature and pressure, it decomposes back to pure oxygen. It is 1.6-fold denser and 10-fold more soluble in water than oxygen. Its half-life at 20 °C is 40 min, which means it cannot be stored [1,2]. Ozone liquefies at temperatures below −111.9 °C and solidifies at −192.7 °C.

Even though ozone is not itself a radical, it is the third most potent oxidant (E_5 12.076 V). The O_3_ molecule breaks down into a diatomic oxygen molecule and active atomic oxygen, which is a very powerful oxidant. It disrupts bacterial cell walls by reacting with polyunsaturated fatty acids, phospholipids, and proteins containing methionine, cysteine, and histidine in the cell membrane. Modification of the cell membrane disrupts and blocks the enzyme system, leading to secondary DNA damage and, ultimately, bacterial cell death. What is more, it increases the amount of ATP released into tissues, decreases the amount of NADH, and helps to oxidize cytochrome C, improving tissue oxygenation and nutrient supply [3,4,5].

In order to harness the properties of ozone for medical purposes, given that it cannot be stored, it is produced using an ozone generator that passes pure oxygen through a high-voltage gradient of 5 to 13 mV. The final product is a gas mixture containing 95% oxygen and 5% ozone [6]. If there is any air in the mixture, it may lead to the formation of toxic nitrogen dioxide. The generator must have an accurate photometer and calculate ozone concentrations in the mixture accurately. If used incorrectly or in larger quantities, ozone can be toxic and can cause pulmonary complications. Oxidation of proteins, thiols, and amines during the peroxidation of polyunsaturated fatty acids can cause serious damage to the body [7].

Nevertheless, the ability to produce ozone in generators has led to intensive research into the effects of ozone on living organisms and its use in therapy. In general, medical applications, the oxygen–ozone mixture is used at concentrations of 25–75 mg/mL. At 20–30 mg/mL, ozone has an immunostimulating action; at 40–45 mg/mL, it improves tissue oxygenation; and at 60–75 mg/mL, it achieves bactericidal properties [6,8,9].

Ozone therapy can take a variety of forms: dry baths in the oxygen–ozone mixture, dressings with ozone water or ozonated oil, intravenous or intra-articular administration of ozonated infusion fluids or autohemotherapy [3,4]. Many authors acknowledge that ozone therapy should be used as a complementary approach, often in combination with conventional treatment, but not as an alternative. It should be said that ozone therapy, as used currently, is based on very well-understood biochemical, physiological, and pharmacological processes, providing a clear rationale for the therapeutic effects and preventing harmful effects. From a pharmacological point of view, ozone therapy follows the principle of hormesis: it has a high efficacy at lower concentrations, but at higher doses it can be ineffective or even toxic. At low doses, this potent oxidizing agent stimulates endogenous antioxidant activity and the production of interleukins and leukotrienes, alleviating inflammation and pain [6,10]. The bactericidal, virucidal, and fungicidal effects of ozone are used to treat infections in fields including surgery, dermatology, cosmetology, and dentistry, and its controlled application makes for safe treatment [2].

Examples of the use of ozone in general medicine include treatment of back pain, a condition that affects up to 80% of the world’s population [11,12,13]. Dr. A. Balkanyi in Zurich is believed to have been the first to treat patients with pain caused by tendinitis and myofascial pain by injecting small amounts of ozone [14,15,16]. Subsequently, ozone was used by other therapists to treat acute and chronic polyarthritis, diseases of the joints, and Morton’s neuroma with intra-articular or periarticular administration of 5–10 mL of an ozone–oxygen mixture (with ozone concentration ranging from 5 to 15 μg/mL) [17,18,19].

Ozone has also been applied to treat liver cirrhosis, an increasing cause of morbidity and mortality in more developed countries [20], as well as age-related macular degeneration. Given population ageing trends, AMD is a significant problem. Studies have shown that ozone therapy can halt the progression of the disease while improving patients’ visual acuity and well-being. Furthermore, research findings indicate that ozone therapy is safe [21,22,23,24]. In their paper, Borrelli and Bocci suggested that ozone therapy may activate a number of defense mechanisms against ischemic and neurotoxic injury and thus prevent photoreceptor death [24].

Ozone therapy is used in pulmonary diseases. Leroy et al. [25,26,27,28] demonstrated that human exposure to ozone (200 ppb) causes a significant increase in the expression of various genes involved in wound healing. The authors studied nineteen people, with and without asthma, who were exposed to clean air (0 ppb), low (100 ppb), and high (200 ppb) ambient ozone concentrations for four hours in a special chamber. At 20 h after the end of exposure, the subjects underwent bronchoscopy with bronchoalveolar lavage (BAL). The results of the study indicate that the alveolar parenchyma responds to exposure to ozone by activating genes involved in immunoactivation. Processes such as chemotaxis (cell chemotaxis, leukocyte chemotaxis, and granulocyte chemotaxis) and responses to cytokines were enhanced.

**Figure 1:**
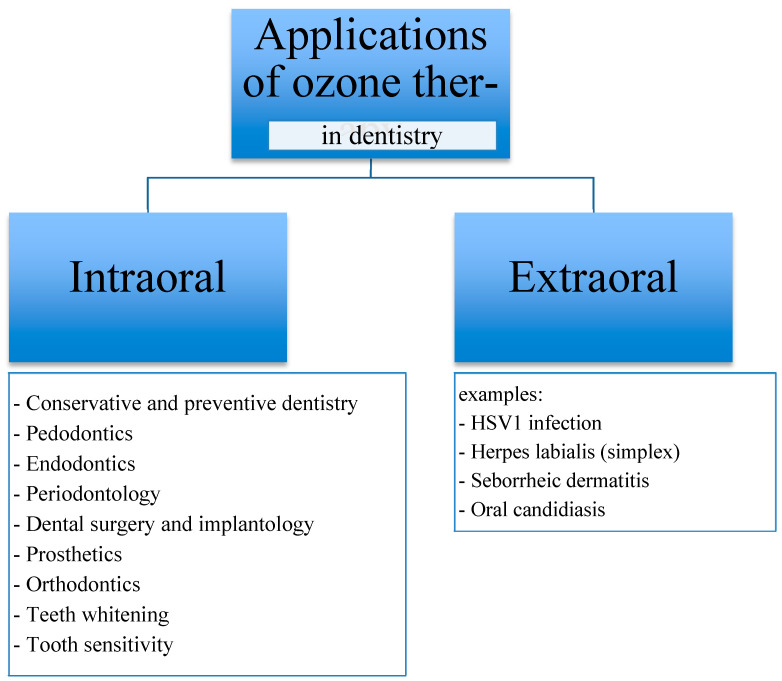
List of intraoral and extraoral applications of ozone therapy in dental specialties.

The use of ozone is also quite common and regarded as safe in the treatment of skin diseases. Ozone has a low penetration potential into cutaneous tissues as it reacts rapidly with polyunsaturated fatty acids and the water of the stratum corneum. This leads to the formation of ROS and lipo-oligopeptides (LOP), which in turn are readily scavenged by skin antioxidants or partially absorbed by the venous and lymphatic capillaries [26,29]. Ozonated oil is one form of ozone therapy in skin diseases. Applied topically, the oil is used in the treatment of wounds, ulcers, burns, cellulitis, abscesses, fistulas, gingivitis, and vaginitis, as well as herpetic and anaerobic infections [30,31,32,33]. Kim et al. suggested that ozonated oil accelerates cutaneous wound healing by increasing the expression of PDGF, TGF-β1, and VEGF [34]. Other studies suggest that ozone exposure is associated with activation of transcription factor NF-κB, which plays a key role in inflammatory response regulation and wound-healing mechanisms [19,20,35,36,37,38]. Ozone exposure has also been observed to reduce the severity of radiodermatitis lesions in cancer patients [39,40,41,42]. Methods of ozone application are summarized in Figure 1.

## 2. Ozone Therapy in Dentistry

Ozone has been successfully used in medicine for many years. With its bactericidal properties against oral bacteria such as: *Streptococcus mutant*, *Streptococcus sobrinus*, *Lactobacillus acidofilus*, *Enterococcus faecalis*, *Peptostreptococcus micros*, *Psudomonas aeruginosa*, *Aggregatibacter actinomycetemcomitans*, *Porphyromonas gingivalis*, *Tannerella forsythia*, *Parvimonas micra*, *Fusobacterium nucleatum*, *Campylobacter sputorum*, *Campylobacter gracilis*, *Eikenella corrodens*, *Helicobacter pylori*, *Candida albicans*, *Candida glabrata* and Herpes viruses [43,44,45,46,47,48,49], it has also found applications in various branches of dentistry. Ozone is used not only in dental treatment and aesthetic procedures such as teeth whitening, but also for disinfecting the waterline of the dental unit and the dental office itself [50,51].

## 3. Ozone Therapy Modalities Used in Dentistry

Ozone therapy modalities used in dentistry include: ozone gas, ozonated water, and ozonated olive oil. Water and olive oil have the capacity to capture and then release ozone, providing for greater control over the procedure than when using ozone in gaseous form. The aforementioned modalities are used alone or in combination depending on the clinical application and the expected results of treatment of the teeth and periodontal tissues [22,23,24,25,26]. The use of ozone in the above-mentioned modalities is not limited to conservative dentistry, but has found applications in many treatments representing various fields, including endodontics, periodontics, surgery [43,44,52,53,54,55,56], and even head and neck oncology [2,7,56].

## 4. Aim of the Study

The aim of this literature review is to analyze the clinical relevance and potential use of ozone in modern dentistry. The research question is geared towards detailing the multiple potential applications of ozone therapy in a range of dental specialties.

## 5. Material and Methods

Based on the available literature, accessed via the PubMed, Google Scholar, Scopus, and EBSCO databases, a detailed search of the electronic literature was performed for 2001–2022. Eligible studies were chosen according to inclusion and exclusion criteria, using keywords: ozone, ozone therapy, therapeutic applications, oxidants, dental disinfectants, oral medicine, physical therapy in dentistry. Studies were selected with a view to diversity, methodological rigor, and research ethics. A multi-step selection process was used to identify reports meeting the selection criteria, which were then subjected to a critical appraisal.

Out of 834 manuscripts, 273 studies were curated, which were used in part to characterize the general use of ozone therapy, to determine its relevance in the elimination of specific bacterial strains, and to describe forms of ozone administration in clinical practice. A total of 70 publications were used in the final consideration. After assessing their quality, they were analyzed to determine the relevance and potential use of ozone in the various aspects of modern dentistry. The papers used in the review represented different types of manuscripts, 70 papers were analyzed, 32 clinical trial reports, 32 review papers, and 6 case reports.

**Figure 2:**
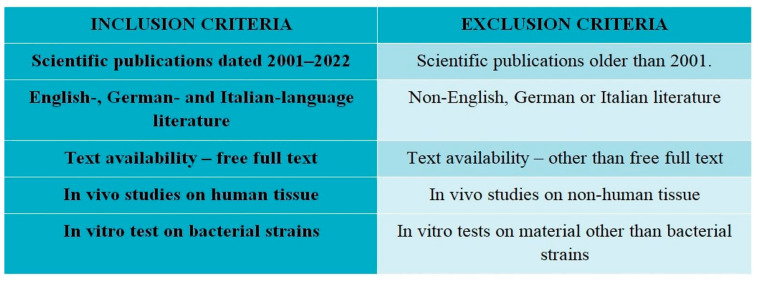
Inclusion and exclusion criteria used in this literature review.

The inclusion and exclusion criteria used in this review are shown in Figure 2.

**Figure 3:**
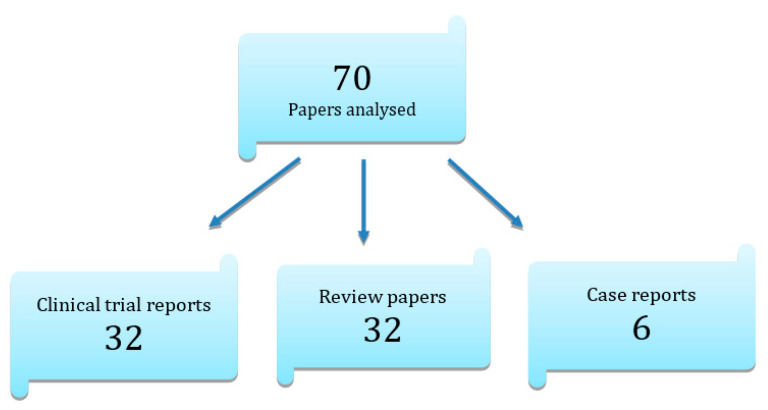
Breakdown of the literature included in the review by manuscript type.

The papers used in the review represented different types of manuscripts, as shown in Figure 3.

## 6. Main Issue

The research question is geared towards detailing the multiple potential applications of ozone therapy in a range of dental specialties.

### 6.1. Conservative Dentistry

Dental caries is a chronic multifactorial disease caused by bacteria that can damage teeth in both children and adults. Caries is widespread in all cultural groups and is one of the most common health conditions affecting the world population (WHO 1990). Treating and preventing dental caries is the remit of restorative dentistry, and in this field, ozone has also been considered as a treatment and prevention modality.

Ozone has been shown to have a toxic effect against some groups of bacteria, and this has given rise to the hope that ozone delivered into a carious lesion can be used to reduce bacterial counts [57]. Control of the bacterial biofilm is one of the pillars of conservative dentistry. The study conducted by M. Nagayoshi demonstrated the effect of ozone on bacterial plaque. After exposing the sample to ozonized water, there was a significant decrease in the number of bacteria including *S. mutans*, the major pathogen of dental caries. During the experiment, a decrease in the viability of bacteria *S. mutans* was noted at 58% after exposure to 0.5 mg/L of ozonated water for 10 s, while using concentrations of 2 mg/L and 4 mg/L, the reduction in bacteria was 100%. Survival of other microorganisms important in the cariogenic process, such as *S. sobrinus*, *S. sanguis*, and *S. salivarius*, was recorded at a similar level to *S. mutans*. The results suggest that ozonated water may be useful in controlling oral infectious microorganisms present in dental plaque [58,59].

In modern dentistry, the use of ozone has been tested as a treatment for cavities of carious origin. One such attempt was undertaken by Aylin Baysan and Edward Lynch. Ozone was applied to root carious lesions. The study showed a statistically significant reduction in the bacterial count in the carious lesion, leading to a change in the disease process to a stage making it possible to conclude that further progression had stopped [60]. Overall, however, research findings are inconclusive. Experiments by other researchers found no effect of ozone therapy on halting or delaying disease progression [57].

### 6.2. Pedodontics

Ozone is also used in pediatric dentistry to disinfect cavities in deep caries. It can be applied in the cavity after the soft, demineralized and infected dentin is removed, which, together with the application of demineralizing substances, helps avoid irreversible pulp damage [48].

### 6.3. Teeth Whitening

The oxidizing properties of ozone have inspired researchers to use it as an alternative bleaching substance for stained teeth [61,62,63]. Tessier et al., showed that the yellowing of tetracycline-stained rat incisors was diminished after applying ozone for a minimum of 3–4 min [6,44]. However, more recent reports point out that the above study should not be regarded as sufficient evidence for the efficacy of ozone in removing these stains, due to the differences in size and chemical composition of rat and human teeth, their pigmentation and the application technique [32].

In a comparison with hydrogen peroxide, ozone was not shown to be a more effective bleaching agent, which may be due to the pH-reducing hydroxyl radicals produced by hydrogen peroxide [6,44,61,64]. A higher pH has been associated with more effective tooth bleaching [61,63].

However, the synergistic effect of ozone on bleaching with simultaneous use of hydrogen peroxide remains debatable. In the study by Naik et al., this effect was not demonstrated [50], while another study, by Al-Omiri et al., showed that the use of 38% hydrogen peroxide together with ozone provided an improved bleaching effect [6]. Even more recent studies have attributed the superior bleaching action of hydrogen peroxide in combination with ozone to the possibility that ozone may produce peroxide, which may contribute to the formation of additional hydroxyl radicals, and this is considered independent of the use of ozone before or after H_2_O_2_ [33].

### 6.4. Endodontics

Endodontics is a field of dentistry that has made remarkable progress in recent years. It has been achieved thanks to modern instruments and innovative materials used in endodontic treatment, as well as new diagnostic methods and the use of magnifying devices. The use of ozone has also played a part. As a gas, ozone can reach inaccessible areas, which are difficult to disinfect during standard chemo-mechanical preparation. These include dentinal tubules, apical delta, anastomoses, lateral canals, accessory canals, and the periapical region [43]. According to research findings, ozonated water is effective as a disinfectant against *E. faecalis*, *Candida albicans*, *Peptostreptococcus micros*, and *Pseudomonas aeruginosa*. [44]. Another study found ozone to be effective against *Streptococcus mutans*, *Candida albicans*, and *Staphylococcus aureus* [65]. Lempe et al. demonstrated 100% efficacy of ozone against *E. faecalis*. What is more, even a single 40 s application of ozone into the root canal lumen resulted in the resolution of symptoms such as swelling, pain, and exudate in the root canal [66,67,68]. The antimicrobial effect of ozone is comparable to that of 2.5% NaOCl, but, unlike NaOCl, ozone is not toxic to tissues during endodontic treatment [69,70,71]. Furthermore, ozone has been proven to penetrate through the apical foramen to reach the surrounding bone, where it promotes bone regeneration and healing [3,72]. Reports of the efficacy of ozone in root canal disinfection were contradicted by the study by Estrela et al. In an in vivo study of infected human root canals, the authors showed that the application of gaseous ozone may not be sufficient to remove *E. faecalis* [71].

### 6.5. Periodontology

**Figure 4:**
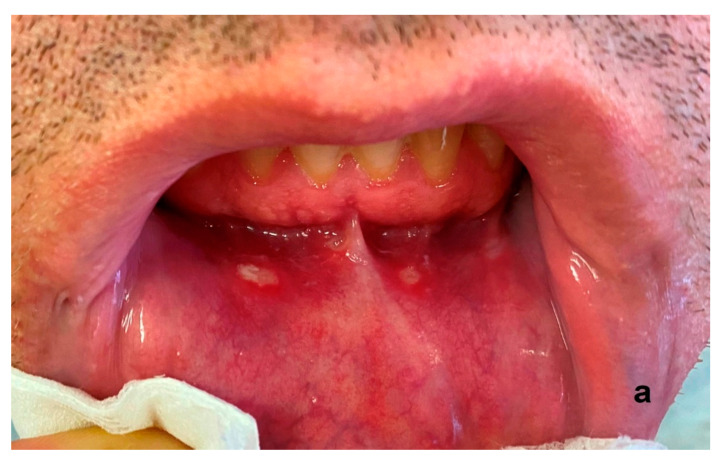

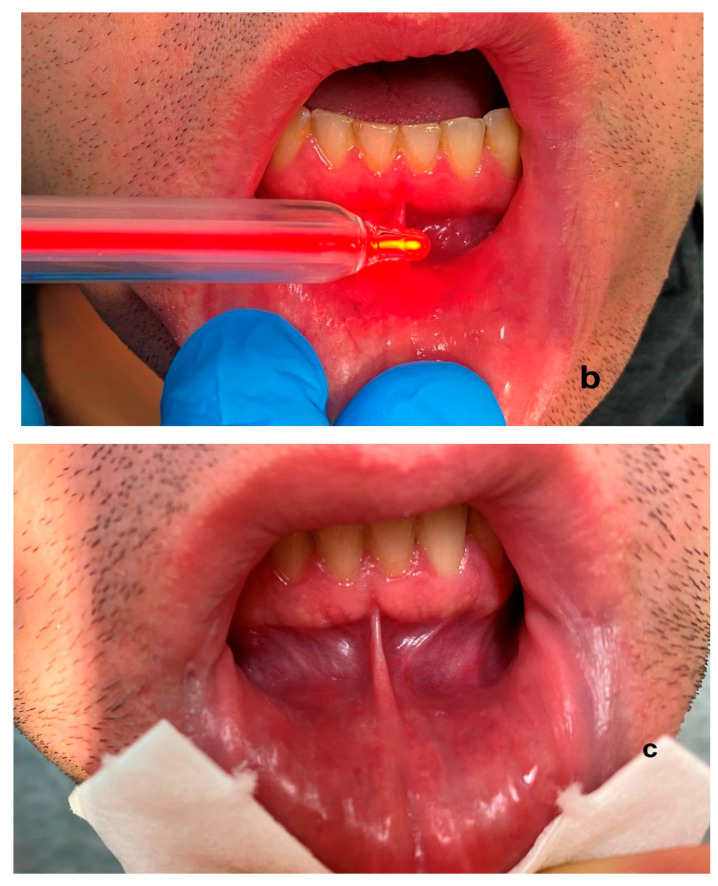
Example of chronic recurrent aphthous ulcers (**a**) before treatment, (**b**) during treatment, (**c**) after three days of ozone therapy. The glass probe of the OzonyTron ozone generator (Mymed) was used (authors’ own photographic documentation).

**Figure 5:**
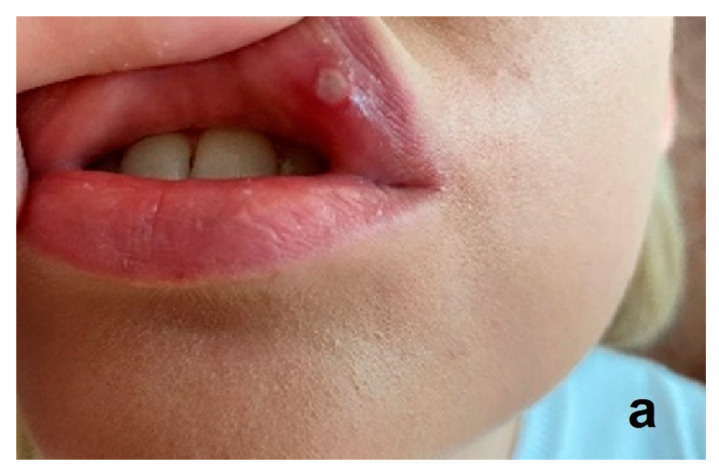

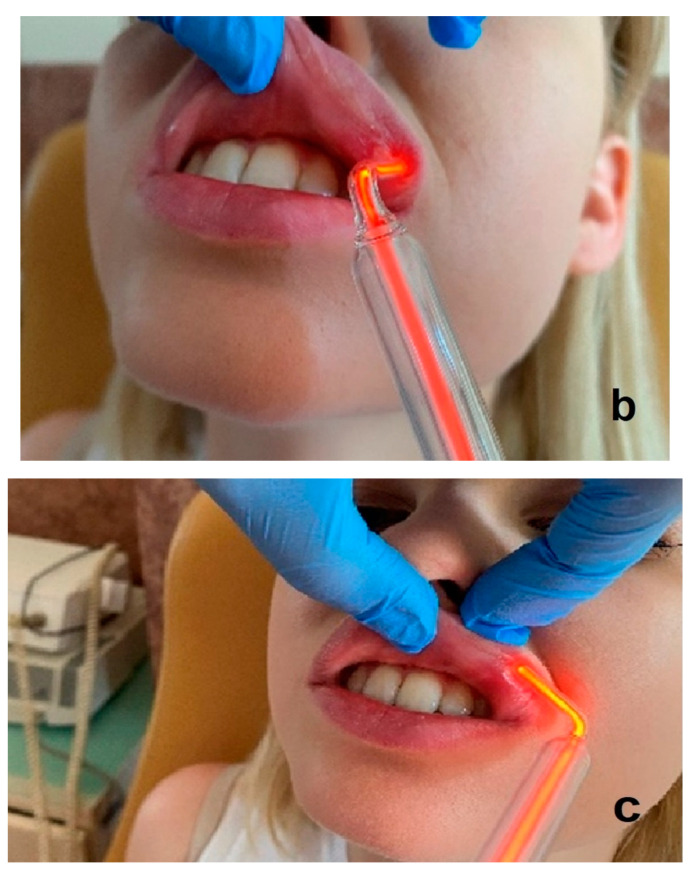
Example of herpes simplex virus on the upper lip mucosa (**a**) before treatment, (**b**) during therapy, (**c**) after three days of ozone therapy. The glass probe of the OzonyTron ozone generator (Mymed) was used (authors’ own photographic documentation).

Ozone has antimicrobial properties, including against anaerobic bacteria that play a role in the pathogenesis of periodontitis. It promotes hemostasis and stimulates the release of growth factors and antioxidant enzymes [73,74,75,76,77,78]. E. Dengizek and colleagues studied the clinical effects of ozone during scaling and root planing (SRP) procedures in the treatment of periodontal disease. They conducted a study on 40 patients with chronic periodontitis. SRP therapy and ozone were used in the experimental group, while the control group received SPR with a placebo. A number of parameters were assessed, including PI, GI, and CAL. Following treatment, all the above-mentioned parameters were similar in both the experimental and control groups, with no statistically significant differences [51]. There are also reports in the literature of positive, clinically significant effects of ozone on periodontal tissues, such as the study by Isler et al. on the effect of ozone in the decontamination of implant surfaces in peri-implantitis [54]. Patients were randomly allocated to either the control group, where sterile saline was used for decontamination of implant surfaces in SRT of peri-implantitis, or to the ozone group, in which ozone was applied additionally. The ozone delivery system used was the OzoneDTA Ozone Generator (DentaTec Dental AS, Hov, Norway). There was a significant difference in PD values between groups at 3-month follow-up, in favor of the ozone group (*p* < 0.05) [54]. The positive effect of ozone therapy in patients with peri-implant mucositis was demonstrated by Mckenna et al., who showed that ozone reduces plaque and delays the development of peri-implant disease [79,80,81]. Diseases of the oral mucosa, such as herpes simplex viral infections, recurrent aphthous stomatitis (RAS), angular cheilitis, or candidiasis can be very difficult for both the patient and the physician. Their chronic and recurrent nature requires specifically designed therapies and often prolonged treatment. The authors of this literature review achieved spectacular success in the treatment of chronic recurrent aphthous ulcers using three two-minute ozone applications (Figure 4a–c), as well as in three applications of ozone therapy, the herpes simplex virus (Figure 5a–c).

### 6.6. Tooth Sensitivity

According to research findings, ozone has the ability to open dentinal tubules. This property is used in the treatment of cervical hypersensitivity, as ozone gas enables the diffusion of calcium and phosphate ions once the tubules are opened. The penetration of these ions, as well as fluoride and zinc ions, is also facilitated by the elimination of bacteria from the dentinal tubules [82,83,84,85,86]. Ozone therapy is also used successfully in the treatment of hypersensitivity induced by tooth preparation in prosthodontics [62,87,88].

### 6.7. Dental Surgery and Implantology

Ozone therapy has been applied in dental surgery to stimulate wound healing and regeneration of damaged tissues [2,6,7,55]. In implantology, ozone has been used to decontaminate the implant prior to implantation. The positive effects of ozone have also been reported in the prevention of postoperative complications such as edema, granulation tissue, dry socket [54], and bone necrosis [55]. With ozone therapy, importantly, patients reported less postoperative pain, even in cases where the use of opioids for analgesia failed to provide relief [7].

The case study by G. Batinjan et al., shows a positive effect of ozone therapy on wound healing after serial extraction and prevention of osteoradionecrosis (ORN) in high-risk patients [55]. The researchers described the case of a high-risk ORN patient treated with ozone in three stages. The first step was preoperative ozone treatment to reduce the number of bacteria in the operational area to a minimum, whereby ozone gas was applied to the relevant section of the gingiva for 40 s using a gingival probe. The next step was alveolus ozonation after extraction. The whole procedure was completed with ozonation after suturing to stimulate circulation and accelerate healing. The patient’s wounds underwent clinical evaluation which clearly showed that healing was without complications or signs of ORN [55].

The effect of ozone on tissue behavior after tooth extraction and severity of postoperative pain was investigated by Jehona Ahmedi et al. [89]. In their study, aimed at evaluating the efficacy of ozone gas in reducing the incidence of dry socket after surgical extraction of mandibular third molars, the influence of the indication for extraction and the difficulty of the procedure on the occurrence of dry socket, the authors proved that in the experimental group, in which ozone was used, dry socket was about five times less likely to occur than in the control group (3.33% and 16.67%, respectively). In the experimental group, the extraction site was treated with ozone for 12 s using Prozone equipment, while the control group received saline solution. After analyzing the results of the treatment, the researchers concluded that the use of O_3_ may reduce the incidence of dry socket and shorten the recovery period after treatment. According to the authors, ozone is recommended for all patients, especially those at risk of developing dry socket after extraction [89,90,91,92,93].

Accelerated healing and absence of significant complications were also reported by Sila Cagri Isler et al. [54]. They conducted a study using ozone gas therapy for decontamination of implant surfaces during surgical regenerative therapy (SRT) in 41 patients with moderate to advanced peri-implantitis. At the end of the 12 month follow-up, they observed significantly superior results in terms of probing depth of the gingival pocket and clinical attachment level of the gingival tissue. They also noticed significantly better bone-defect fill on X-ray in the ozone group. The results indicate that implant surface decontamination with additional ozone therapy in surgical regenerative therapy of peri-implantitis has shown clinical and radiological significance [54].

**Figure 6:**
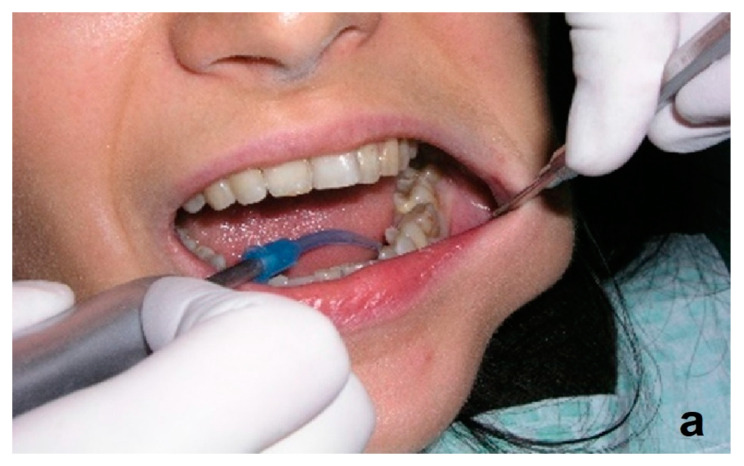

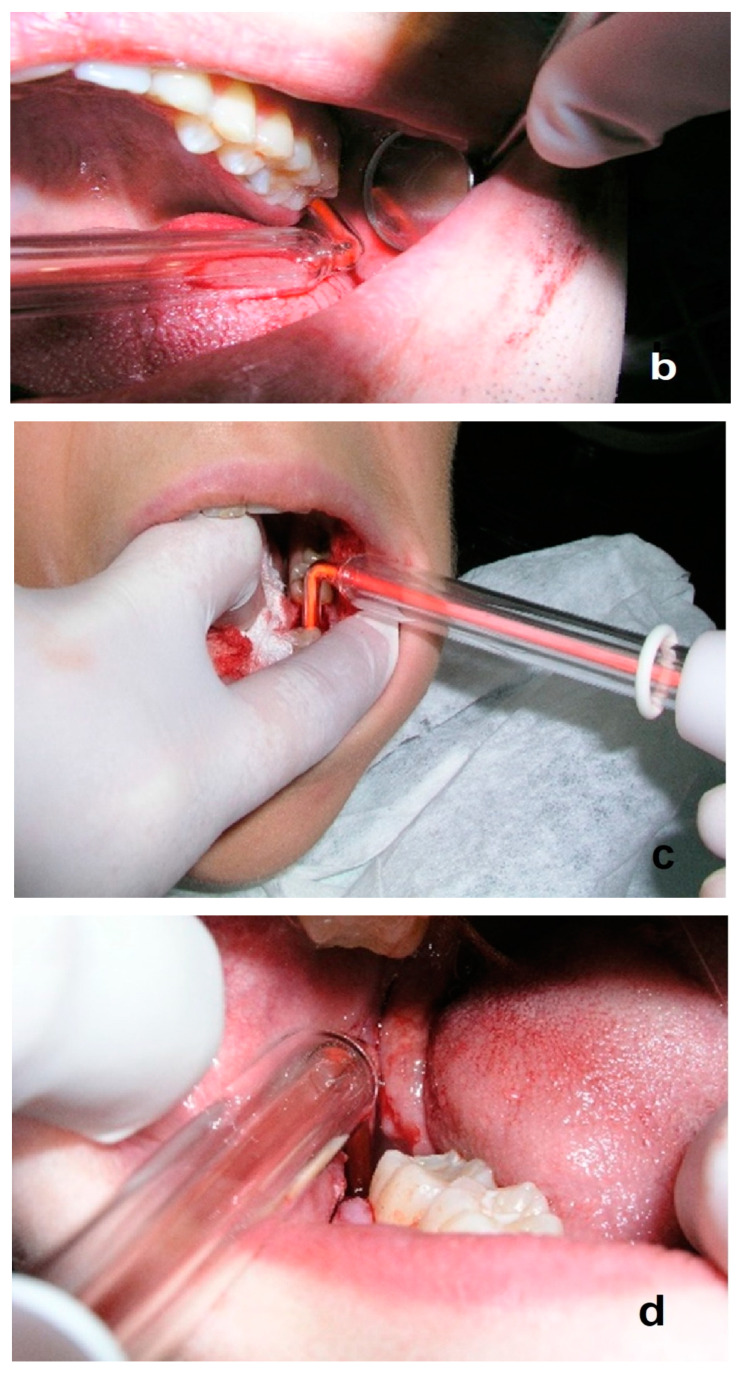
Application of ozone therapy prophylactically before treatment (**a**) and immediately after treatment (**b**–**d**). The glass probe of the OzonyTron ozone generator (Mymed) was used (authors’ own photographic documentation).

The authors of this manuscript apply ozone gas therapy prophylactically before and immediately after surgery to avoid post-extraction complications in the form of edema, trismus, hematoma formation, or raised skin temperature (Figure 6a–d).

### 6.8. Temporomandibular Joint Disorders

The temporomandibular joint (TMJ) is a hinge-type joint that connects the mandible to the temporal bone of the skull. Pathologies within this joint comprise a large group of disease entities of different etiologies. The most common symptoms of disorders in the TMJ region include pain and limited range of motion, affecting quality of life [94,95,96,97]. Daif conducted a study on the efficacy of ozone therapy in TMJ disorders with two randomly assigned groups. One group was treated with an ozone injection into the superior joint space, and the other group was treated with non-steroidal anti-inflammatory drugs. The study demonstrated that the ozone injection contributed to either complete recovery or significant improvement of the internal derangement of the temporomandibular joint in 87% of the patients [88,98,99,100].

## 7. Conclusions

In recent years, ozone therapy has been the subject of much research, making it possible to apply ozone in specialized dental treatments and supporting the use of ozone therapy with objective findings.

Despite a body of evidence demonstrating the safety and positive outcomes of ozone therapy, many clinicians still treat the use of ozone as an alternative, additional and sometimes uncertain modality. The studies carried out in recent years and their findings provide evidence for the significant medical potential of ozone and encourage further research aimed not only at finding new applications, but also at reconfirming and verifying those already known. Ozone therapy is used mainly as an adjunct to the primary clinical or pharmacological treatment. In some cases of oral mucosal disease, it has proven effective as a primary therapy. During the literature analysis, it was noted that ozone therapy in dentistry is a subject of ongoing research, and the results are not always consistent. The multitude of literature on the applications of ozone in dentistry reflects the search for its undiscovered potential as an intraoral and extraoral physical therapy modality in dentistry.

## Data Availability

Not applicable

## References

[1] Saini R. (2011). Ozone therapy in dentistry: A strategic review. J. Nat. Sci. Biol. Med..

[2] Nogales C.G., Ferrari P.H., Kantorovich E.O., Lage-Marques J.L. (2008). Ozone therapy in medicine and dentistry. J. Contemp. Dent. Pract..

[3] Elvis A.M., Ekta J.S. (2011). Ozone therapy: A clinical review. J. Nat. Sci. Biol. Med..

[4] Aydogan S., Artis A.S., Artis A.S. (2012). How Ozone Treatment Affects Erythrocytes. Haemodynamics-New Diagnostic and Therapeutic Approaches.

[5] Borrelli E., Monte A., Bocci V. (2015). ReseArch. article oxygen ozone therapy in the integrated treatment of chronic ulcer: A case series report. Int. J. Rec. Sci. Res..

[6] Suh Y., Patel S., Kaitlyn R., Gandhi J., Joshi G., Smith N.L., Khan S.A. (2019). Clinical utility of ozone therapy in dental and oral medicine. Med. Gas Res..

[7] Oldoini G., Frabattista G.R., Saragoni M., Cosola S., Giammarinaro E., Genovesi A.M., Marconcini S. (2020). Ozone Therapy for Oral Palatal Ulcer in a Leukaemic Patient. Eur. J. Case Rep. Intern. Med..

[8] Louw A., Diener I., Butler D.S., Puentedura E.J. (2011). The Effect of Neuroscience Education on Pain, Disability, Anxiety, and Stress in Chronic Musculoskeletal Pain. Arch. Phys. Med. Rehabil..

[9] Apkarian A.V., Baliki M.N., Geha P.Y. (2009). Towards a theory of chronic pain. Prog. Neurobiol..

[10] Devon I., Rubin M.D. (2007). Epidemiology and Risk Factors for Spine Pain. Neurol. Clin..

[11] Braidy N., Izadi M., Sureda A., Jonaidi-Jafari N., Banki A., Nabavi S.F., Nabavi S.M. (2018). Therapeutic relevance of ozone therapy in degenerative diseases: Focus on diabetes and spinal pain. Cell. Physiol..

[12] Bocci V., Pogni R. (2001). Oxygen-ozone in Orthopaedics: EPR Detection of Hydroxyl Free Radicals in Ozone-Treated ‘NucleusPulposus’ Material. Riv. Neuroradiol..

[13] Legier L. (2005). Treatment of chronic low back pain incorporating active patient participation and chiropractic: A retrospective case report. J. Chiropr. Med..

[14] Hidalgo-Tallón F.J., Torres-Morera L.M., Baeza-Noci J., Carrillo-Izquierdo M.D., Pinto-Bonilla R. (2022). Updated Review on Ozone Therapy in Pain Medicine. Front. Physiol..

[15] Ma L., Yao M. (2022). Safety and Efficacy of CT-Guided Pulsed Radiofrequency Combined with Steroid and Ozone Injection-Treated Cervical 3-8 Herpes Zoster Neuralgia Using a Posterior and Upper Quarter of the Cervical Foramina Puncture Approach. J. Pain Res..

[16] Sagai M., Bocci V. (2011). Mechanisms of Action Involved in Ozone Therapy: Is healing induced via a mild oxidative stress?. Med. Gas Res..

[17] Bocci V., Borrelli E., Zanardi I., Travagli V. (2015). The usefulness of ozone treatment in spinal pain. Drug Des. Dev. Ther..

[18] Steppan J., Meaders T., Muto M., Murphy K.J. (2010). A Meta-analysis of the Effectiveness and Safety of Ozone Treatments for Herniated Lumbar Discs. J. Vasc. Interv. Radiol..

[19] Hidalgo-Tallón F.J., Torres L.M. (2013). Ozonoterapia en medicina del dolor. Revisión. Rev. Soc. Esp. Dolor.

[20] Tsochatzis E., Bosch J., Burroughs A. (2014). Liver cirrhosis. Lancet.

[21] Bocci V. (2013). Visual Improvement Following Ozonetherapy in Dry Age Related Macular Degeneration; a Review. Med. Hypothesis Discov. Innov. Ophthalmol..

[22] Bocci V., Diadori A. (2003). Linee guida per alcune pathologie oculari. Riv. Ital. Ossigeno-Ozonoterapia.

[23] Borrelli E., Diadori A., Zalaffi A., Bocci V. (2012). Effects of major ozonated autohemotherapy in the treatment of dry age related macular degeneration: A randomized controlled clinical study. Int. J. Ophthalmol..

[24] Bocci V.A. (2006). Scientific and medical aspects of ozone therapy. State of the art. A review. Arch. Med. Res..

[25] Leroy P., Tham A., Wong H., Tenney R., Chen C., Stiner R., Balmes J.R., Paquet A.C., Arjomandi M. (2015). Inflammatory and repair pathways induced in human bronchoalveolar lavage cells with ozone inhalation. PLoS ONE.

[26] Smith N.L., Wilson A.L., Gandhi J., Vatsia S., Khan S.A. (2017). Ozone therapy: An overview of pharmacodynamics, current research, and clinical utility. Med. Gas Res..

[27] Bocci V. (2007). The case for oxygen-ozonetherapy. Br. J. BioMed. Sci..

[28] De Sire A., Agostini F., Lippi L., Mangone M., Marchese S., Cisari C., Bernetti A., Invernizzi M. (2021). Oxygen-Ozone Therapy in the Rehabilitation Field:State of the Art on Mechanisms of Action, Safety andEffectiveness in Patients with Musculoskeletal Disorders. Biomolecules.

[29] Bocci V. (2005). OZONE A New Medical Drug.

[30] Żukowski P., Maciejczyk M., Waszkiel D. (2018). Sources of free radicals and oxidative stress in the oral cavity. Arch. Oral Biol..

[31] Di Mauro R., Cantarella G., Bernardini R., Di Rosa M., Barbagallo I., Distefano A., Longhitano L., Vicario N., Nicolosi D., Lazzarino G. (2019). The Biochemical and Pharmacological Properties of Ozone: The Smell of Protection in Acute and Chronic Diseases. Int. J. Mol. Sci..

[32] Brusselle G.G., Koppelman G.H. (2022). Biologic Therapies for Severe Asthma. N. Engl. J. Med..

[33] Tonnel A.B., Gosset P., Tillie-Leblond I. (2001). Characteristics of the Inflammatory Response in Bronchial Lavage Fluids from Patients with Status asthmaticus. Int. Arch. Allergy Immunol..

[34] Kim H.S., Noh S.U., Han Y.W., Kim K.M., Kang H., Kim H.O., Park Y.M. (2009). Therapeutic effects of topical application of ozone on acute cutaneous wound healing. J. Korean Med. Sci..

[35] Al-Dalain S.M., Martinez G., Candelario-Jalil E., Menendez S., Re L., Giuliani A., Leon O.S. (2001). Ozone treatment reduces markers of oxidative and endothelial damage in an experimental diabetes model in rats. Pharm. Res..

[36] Uruno A., Yagishita Y., Yamamoto M. (2015). The Keap1-Nrf2 system and diabetes mellitus. Arch. Biochem. Biophys..

[37] Lim A.K.H. (2014). Diabetic nephropathy—Complications and treatment. Int. J. Nephrol. Renovasc. Dis..

[38] Janic B., Umstead T.M., Phelps D.S., Floros J. (2005). Modulatory effects of ozone on thp-1 cells in response to sp-a stimulation. Am. J. Physiol. Lung. Cell. Mol. Physiol..

[39] Valacchi G., van der Vliet A., Schock B.C., Okamoto T., Obermuller-Jevic U., Cross C.E., Packer L. (2002). Ozone exposure activates oxidative stress responses in murine skin. Toxicology.

[40] Kushmakov R., Gandhi J., Seyam O., Jiang W., Joshi G., Smith N.L., Khan S.A. (2018). Ozone therapy for diabetic foot. Med. Gas Res..

[41] Bocci V., Borrelli E., Travagli V., Zanardi I. (2009). The ozone paradox: Ozone is a strong oxidant as well as a medical drug. Med. Res. Rev..

[42] Jordan L., Beaver K., Foy S. (2002). Ozone treatment for radiotherapy skin reactions: Is there an evidence base for practice?. Eur. J. Oncol. Nurs..

[43] Domb W.C. (2014). Ozone Therapy in Dentistry. Interv. Neuroradiol..

[44] Naik S.V., Rajeshwari K., Kohli S., Zohabhasan S., Bhatia S. (2016). Ozone- A Biological Therapy in Dentistry- Reality or Myth?. Open Dent. J..

[45] Gupta G., Mansi B. (2012). Ozone therapy in periodontics. J. Med. Life..

[46] Song M., Zeng Q., Xiang Y., Gao L., Huang J., Huang J., Wu K., Lu J. (2018). The antibacterial effect of topical ozone on the treatment of MRSA skin infection. Mol. Med. Rep..

[47] Longbottom C., Ekstrand K., Zero D., Kambara M. (2009). Novel preventive treatment options. Monogr. Oral Sci..

[48] Gaines S., Serota K.S. (2005). The new frontier: Minimally invasive dentistry and ozone aerotheraphy. Oral Health.

[49] Liu J., Zhang P., Tian J., Li L., Li J., Tian J.H., Yang K. (2015). Ozone therapy for treating foot ulcers in people with diabetes. Cochrane Database Syst. Rev..

[50] Reddy S., Reddy N., Dinapadu S., Reddy M., Pasari S. (2013). Role of Ozone Therapy in Minimal Intervention Dentistry and Endodontics—A Review. J. Int. Oral Health.

[51] Seydanur Dengizek E., Serkan D., Abubekir E., Aysun Bay K., Onder O., Arife C. (2019). Evaluating clinical and laboratory effects of ozone in non-surgical periodontal treatment: A randomized controlled trial. J. Appl. Oral Sci..

[52] Onder C., Kurgan Ş., Altıngöz S.M., Bağış N., Uyanık M., Serdar M.A., Kantarcı A., Günhan M. (2017). Impact of non-surgical periodontal therapy on saliva and serum levels of markers of oxidative stress. Clin. Oral Investig..

[53] Huth K.C., Quirling M., Lenzke S., Paschos E., Kamereck K., Brand K., Hickel R., Ilie M. (2011). Effectiveness of ozone against periodontal pathogenic microorganisms. Eur. J. Oral Sci..

[54] Isler S.C., Unsal B., Soysal F., Ozcan G., Peker E., Karaca I.R. (2018). The effects of ozone therapy as an adjunct to the surgical treatment of peri-implantitis. J. Periodontal Implant. Sci..

[55] Batinjan G., Zore I.F., Vuletić M., Rupić I. (2014). The use of ozone in the prevention of osteoradionecrosis of the jaw. Saudi Med. J..

[56] Jacobson A.S., Buchbinder D., Hu K., Urken M.L. (2010). Paradigm shifts in the management of osteoradionecrosis of the mandible. Oral Oncol..

[57] Rickard G.D., Richardson R., Johnson T., McColl D., Hooper L. (2004). Ozone therapy for the treatment of dental caries. Cochrane Database Syst. Rev..

[58] Nagayoshi M., Fukuizumi T., Kitamura C., Yano J., Terashita M., Nishihara T. (2004). Efficacy of ozone on survival and permeability of oral microorganisms. Oral Microbiol. Immunol..

[59] Rumbaugh K.P., Sauer K. (2020). Biofilm dispersion. Nat. Rev. Microbiol..

[60] Baysan A., Lynch E. (2004). Effect of ozone on the oral microbiota and clinical severity of primary root caries. Am. J. Dent..

[61] Zanjani V., Ghasemi A., Torabzadeh H., Jamali M., Razmavar S. (2015). Bleaching effect of ozone on pigmented teeth. Dent. Res. J..

[62] Azarpazhooh A., Limeback H. (2008). The application of ozone in dentistry: A systematic review of literature. J. Dent..

[63] Bengel W.M. (2003). Digital photography and the assessment of therapeutic results after bleaching procedures. J. Esthet. Restor. Dent..

[64] AL-Omiri K., Al Nazeh A., KIelbassa A., Lynch E. (2018). Randomized controlled clinical trial on bleaching sensitivity and whitening efficacy of hydrogen peroxide versus combinations of hydrogen peroxide and ozone. Sci. Rep..

[65] Cardoso M.G., de Oliveira L.D., Koga-Ito C.Y., Jorge A.O.C. (2008). Effectiveness of ozonated water on Candida albicans, Enterococcus faecalis, and endotoxins in root canals. Oral Surg. Oral Med. Oral Pathol. Oral Radiol. Endodontol..

[66] Lynch E. (2009). Comment on “The application of Ozone in dentistry: A systematic review of the literature”. J. Dent..

[67] Elkarim I., Kennedy J., Hussey D. (2007). The antimicrobial effects of root canal irrigation and medication. Oral Surg. Oral Med. Oral Pathol..

[68] Bachanek T., Bieżanek T., Strycharz M., Chałas R., Paluch-Oleś J. (2008). Ozone influence on elimination of bacteria from the root canals—Preliminary observations. Pol. J. Environ. Stud..

[69] Estrela C., Estrela C.R.A., Decurcio D., Hollanda A., Silva J. (2007). Antimicrobial efficacy of ozonated water, gaseous ozone, sodium hypochlorite and chlorhexidine in infected human root canals. Int. Endod. J..

[70] Huth K.C., Quirling M., Maier S., Kamereck K., Alkhayer M., Paschos E., Welsch U., Miethke T., Brand K., Hickel R. (2009). Effectiveness of ozone against endodontopathogenic microorganisms in a root canal biofilm model. Int. Endod. J..

[71] Baysan A., Lynch E. (2005). The use of ozone in dentistry and medicine. Prim. Dent. Care.

[72] Lubojanski A., Dobrzynski M., Nowak N., Rewak-Soroczynska J., Sztyler K., Zakrzewski W., Dobrzynski W., Szymonowicz M., Rybak Z., Wiglusz K. (2021). Application of Selected Nanomaterials and Ozone in Modern Clinical Dentistry. Nanomaterials.

[73] Eick S., Tigan M., Sculean A. (2012). Effect of ozone on periodontopathogenic species--an in vitro study. Clin. Oral Investig..

[74] Kronenberg O., Lussi A., Ruf S. (2009). Preventive effect of ozone on the development of white spot lesions during multibracket appliance therapy. Angle Orthod..

[75] Dai Z., Liu M., Ma Y., Cao L., Xu H.H., Zhang K., Bai Y. (2019). Effects of Fluoride and Calcium Phosphate Materials on Remineralization of Mild and Severe White Spot Lesions. BioMed. Res. Int..

[76] Almaz M.E., Sönmez I.Ş. (2015). Ozone therapy in the management and prevention of caries. J. Formos. Med. Assoc..

[77] Ozdemir H., Toker H., Balcı H., Ozer H. (2013). Effect of ozone therapy on autogenous bone graft healing in calvarial defects: A histologic and histometric study in rats. J. Periodontal Res..

[78] Jan A., Sandor G.K., Brkovic B.B., Peel S., Evans A.W., Clokie C.M. (2009). Effect of hyper- baric oxygen on grafted and nongrafted calvarial critical-sized defects. Oral Surg. Oral Med. Oral Pathol. Oral Radiol. Endod..

[79] McKenna D.F., Borzabadi-Farahani A., Lynch E. (2013). The effect of subgingival ozone and/or hydrogen peroxide on the development of peri-implant mucositis: A double-blind randomized controlled trial. Int. J. Oral Maxillofac Implant..

[80] Megighian G.D., Lynch E. (2004). The clinical experience in private general dental practice in Italy. Ozone: The Revolution in Dentistry.

[81] Dukić W., Dukić O.L., Milardović S. (2009). The influence of Healozone on micro leakage and fissure penetration of different sealing materials. Coll. Antropol..

[82] Maiya A. (2011). Applications of Ozone in Dentistry. Int. J. Clin. Dent. Sci..

[83] Martínez-Sánchez G., Saied M., Al-Dalain Menéndez S., Re L., Giuliani A., Candelario-Jalil E., Álvarez H., Fernández-Montequín J.I., León O.S. (2005). Therapeutic efficacy of ozone in patients with diabetic foot. Eur. J. Pharmacol..

[84] Malik T., Kaura S., Kakria P. (2020). Dental ozone: A boon for dentistry. Indian J. Dent. Sci..

[85] Bhateja S. (2012). The miraculous healing therapy “Ozone therapy” in dentistry. Indian J. Dent..

[86] Maiorana C., Grossi G.B., Garramone R.A., Manfredini R., Santoro F. (2013). Do ultrasonic dental scalers interfere with implantable cardioverter defibrillators? An in vivo investigation. J. Dent..

[87] Bin-Shuwaish M.S. (2016). Effects and Effectiveness of Cavity Disinfectants in Operative Dentistry: A Literature Review. J. Contemp. Dent. Pract..

[88] Stübinger S., Sader R., Filippi A. (2006). The use of ozone in dentistry and maxillofacial surgery: A review. Quintessence Int..

[89] Ahmedi J., Ahmedi E., Sejfija O., Agani Z., Hamiti V. (2016). Efficiency of gaseous ozone in reducing the development of dry socket following surgical third molar extraction. Eur. J. Dent..

[90] Boch T., Tennert C., Vach K., Al Ahmad A., Hellwig E., Polydorou O. (2016). Effect of gaseous ozone on Enterococcus faecalis biofilm an in vitro study. Clin. Oral Investig..

[91] Loncar B., Mravak Stipetic M., Matosevic D., Tarle Z. (2009). Ozone application in dentistry. Arch. Med. Res..

[92] Agapov V.S., Shulakov V.V., Fomchenkov N.A. (2001). Ozone therapy of chronic mandibular osteomyelitis. Stomatologiia.

[93] Moezizaden M. (2013). Future of dentistry, nanodentistry, ozone therapy and tissue engineering. J. Dev. Biol. Tissue Eng..

[94] Meghan K., MacBarb R.F., Wong M.E., Athanasiou K.A. (2013). Temporomandibular JoInt. Disorders: A Review of Etiology. Clinical Management and Tissue Engineering Strategies. J. Oral Maxillofac Implant..

[95] Valesan L.F., Da-Cas C.D., Réus J.C., Denardin AC S., Garanhani R.R., Bonotto D., Januzzi E., Mendes de Souza B.D. (2021). Prevalence of temporomandibular joInt. disorders: A systematic review and meta-analysis. Clin. Oral Investig..

[96] Goncalves D.A., Fabbro A.L., Campos J.A., Bigal M.E., Speciali J.G. (2010). Symptoms of temporomandibular disorders in the population: An epidemiological study. J. Orofac. Pain..

[97] Klasser G.D., Greene C.S. (2009). Oral appliances in the management of temporomandibular disorders. Oral Surg. Oral Med. Oral Pathol. Oral Radiol. Endod..

[98] Daif E.T. (2012). Role of intra-articular ozone gas injection in the management of internal derangement of the temporomandibular joint. Oral Surg. Oral Med. Oral Pathol. Oral Radiol..

[99] Bocci V. (2004). Ozone as Janus: This controversial gas can be either toxic or medically useful. Mediat. Inflamm..

[100] Larheim T.A. (2005). Role of Magnetic Resonance Imaging in the Clinical Diagnosis of the Temporomandibular Joint. Cells Tissues Organs.

